# A pilot trial of intravital microscopy in the study of the tumor vasculature of patients with peritoneal carcinomatosis

**DOI:** 10.1038/s41598-021-84430-3

**Published:** 2021-03-02

**Authors:** Emmanuel M. Gabriel, Minhyung Kim, Daniel T. Fisher, Catherine Mangum, Kristopher Attwood, Wenyan Ji, Debabrata Mukhopadhyay, Sanjay P. Bagaria, Matthew W. Robertson, Tri A. Dinh, Keith L. Knutson, Joseph J. Skitzki, Michael B. Wallace

**Affiliations:** 1 Department of Surgery, Section of Surgical Oncology, Mayo Clinic Florida, 4500 San Pablo Road, Jacksonville, FL 32224 USA; 2 Department of Immunology, Roswell Park Comprehensive Cancer Center, Buffalo, NY USA; 3 Department of Biostatistics, Roswell Park Comprehensive Cancer Center, Buffalo, NY USA; 4 Department of Molecular Biology, Mayo Clinic, Jacksonville, FL USA; 5 Department of Gynecological Oncology, Mayo Clinic, Jacksonville, FL USA; 6 Department of Immunology, Mayo Clinic, Jacksonville, FL USA; 7 Department of Surgical Oncology, Roswell Park Comprehensive Cancer Center, Buffalo, NY USA; 8 Department of Gastroenterology, Mayo Clinic, Jacksonville, FL USA

**Keywords:** Cancer imaging, Gastrointestinal cancer, Gynaecological cancer

## Abstract

Aberrancies in the tumor microvasculature limit the systemic delivery of anticancer agents, which impedes tumor response. Using human intravital microscopy (HIVM), we hypothesized that HIVM would be feasible in patients with peritoneal carcinomatosis (PC). During cytoreductive surgery with hyperthermic intraperitoneal chemotherapy for PC, HIVM was performed in both tumor and non-tumor areas. The primary outcome was HIVM feasibility to measure vessel characteristics. We secondarily evaluated associations between HIVM vessel characteristics and oncologic outcomes (RECIST response to neoadjuvant therapy and disease-specific survival). Thirty patients with PC were enrolled. Nineteen patients (63.3%) received neoadjuvant therapy. HIVM was feasible in all patients. Compared to non-tumor (control) areas, PC areas had a lower density of functional vessels, higher proportion of non-functional vessels, smaller lumenal diameters, and lower blood flow velocity. Qualitative differences in these vessel characteristics were observed among patients who had partial response, stable disease, or progressive disease after receiving neoadjuvant therapy. However, no statistically significant relationships were found between HIVM vessel characteristics and oncologic outcomes. These novel findings comprise the first-in-human, real-time evidence of the microscopic differences between normal and tumor-associated vessels and form the basis for our larger, ongoing clinical trial appropriately powered to determine the clinical utility of HIVM (NCT03823144).

## Introduction

Peritoneal carcinomatosis (PC) occurs in several malignancies, including those of gastrointestinal (GI) origin (most commonly the appendix and colon/rectum), the genitourinary (GU) tract (most commonly ovarian), primary peritoneal mesothelioma, and to a lesser extent from sarcoma (sarcomatosis) and stomach origin. PC is estimated to affect 13–17% of patients with GI malignancies and as much as 34% of patients with GU maligancies^1–3^. Current treatments for PC include systemic chemotherapy/immunotherapy and regional therapies, namely cytoreductive surgery with hyperthermic peritoneal chemotherapy (CRS-HIPEC). CRS-HIPEC combined with systemic chemotherapy has significant oncologic benefits for each of these surface malignancies^4–7^. Despite these advances in multimodal therapies, however, current treatments for PC still generate heterogeneous responses, and some patients may experience limited to no response from CRS-HIPEC or systemic chemotherapy. This represents a broad gap in the current knowledge and treatment of PC.

HIPEC is directly administered and circulated within the peritoneal cavity during cytoreductive surgery. The intraperitoneal chemotherapy directly penetrates the peritoneal surface of the internal abdominal wall and the external surfaces of the intra-abdominal organs to a depth of a few millimeters (mm)^8,9^. Thus, it is most effective for microscopic disease after the gross debulking of larger PC implants. In contrast, intravenously (IV) administered chemotherapy relies on both the normal systemic vasculature and the tumor microvasculature to deliver the drug to the target tumor. Systemic chemotherapy is often given as a neoadjuvant approach, and patients who have good response may be candidates for CRS-HIPEC.

For bulky tumors, systemic chemotherapy may generate antitumor responses because it is expected to reach the entire tumor, unlike HIPEC which only acts at the tumor surface. While the delivery and distribution of drug to the tumor is assumed to be similar among different patients, this may not be the case. Direct, real-time observation of tumor-associated blood vessels in humans has shown that different patients have varying proportions of functional and non-functional tumor-associated vessels^10^. In a pilot study using intravital microscopy (IVM) to observe melanoma-associated blood vessels in human subjects, up to 50% of these vessels were non-functional in that they did not support blood flow. This observation has critical implications for systemically delivered cancer therapies. The most successful cancer therapies require adequate distribution to the tumor, or else they are rendered ineffective^11–13^. Animal models have provided further evidence that the aberrant structure and function of tumor vessels contributes to decreased drug efficacy^14–16^. Thus, while it is important that cancer therapeutics be effective at the cellular and molecular levels, it is also imperative that these drugs can be successfully delivered to and distributed within the tumor targets via the systemic and tumor circulation.

To date, there have been no studies investigating the real-time observation of PC-associated blood vessels using human IVM (HIVM). While there are many biologic differences between PC of GI/GU origins and melanoma, which has been previously studied using HIVM^10^, PC is anatomically similar to melanoma in that they are both surface malignancies. Thus, PC and its associated vasculature can theoretically be analyzed using HIVM. Differences in PC-associated vessel structure and function may contribute to the heterogeneous responses observed in PC patients who receive neoadjuvant chemotherapy. The characterization of PC-associated blood flow parameters, including vessel diameter, blood flow rates, and vessel density (for both functional and nonfunctional blood vessels), could have profound utility in predicting drug uptake and therefore response in patients with PC. Thus, the overall purpose of this clinical trial was to characterize the tumor vasculature associated with PC in real time using HIVM. The primary objective was to determine the feasibility of HIVM in PC patients at the time of CRS-HIPEC. We hypothesized that HIVM would be feasible in the study of PC-associated blood vessels. Secondary objectives included exploratory analyses of the associations between (1) HIVM tumor vessel observations and response to neoadjuvant therapy and (2) HIVM tumor vessel observations and oncologic survival outcomes.

## Results

### Patient demographics, tumor-specific characteristics, and treatment outcomes

Between January 1, 2018 and December 31, 2019, a total of 45 patients were accrued, of which 30 were enrolled into this study. Patient and tumor-specific characteristics are shown in Table 1. Most patients had PC of either appendiceal (40%) or ovarian (40%) origin. Nearly two-thirds of the cohort (19 patients, 63.3%) received some form of neoadjuvant therapy. Only 4 patients (13.3%) received surgery without HIPEC. For 3 of these patients, each had received neoadjuvant chemotherapy and achieved a partial radiographic response and there was no detectable disease at the time of surgery. Therefore, HIPEC was aborted to minimize complications from cytoreduction, including anastomotic leak from bowel anastomosis. The intra-operative risk–benefit analysis for performing HIPEC was supported by a recent randomized clinical trial showing that HIPEC was not beneficial to survival outcomes for patients with low PCI scores, but increased the risks of complications^17^. For the fourth patient, the PCI score was 30 and the procedure was aborted due to patient instability during the procedure. This patient did not receive neoadjuvant chemotherapy because the pre-operative diagnosis was consistent with disseminated peritoneal adenomucinous (DPAM) originating from a low grade appendiceal mucinous neoplasm (LAMN). However, intra-operative exploration revealed that the disease was unresectable, and pathologic frozen section analysis had upgraded the diagnosis to an appendiceal adenocarcinoma.

**Table 1:** Patient, tumor, and treatment-related variables.

Variable	N (%)
**Age (years)**	
Mean (std)	61.4 (13.6)
**Sex**	
Female	23 (76.7%)
Male	7 (23.3%)
**Body mass index (BMI)**	
Mean (std)	27.7 (5.7)
**Race**	
Asian	1 (3.3%)
Hispanic	2 (6.7%)
White	27 (90.0%)
**Smoking history**	
Current	1 (3.3%)
Former	6 (20.0%)
Never	23 (76.7%)
**Diabetes**	
No	28 (93.3%)
Yes	2 (6.7%)
**Histologic origin**	
Appendix	12 (40.0%)
Ovarian	12 (40.0%)
Other	6 (20.0%)
**Histologic subtype**	
Adenocarcinoma	13 (43.3%)
Serous carcinoma	11 (36.7%)
Other^a^	6 (20.0%)
**Grade**	
Low	8 (28.6%)
Intermediate	3 (10.7%)
High	16 (57.1%)
De-differentiated (liposarcoma)	1 (3.6%)
**Recurrence**	
No	22 (73.3%)
Yes	8 (26.7%)
**Previous abdominal surgery**	
No	6 (20.0%)
Yes^b^	24 (80.0%)
**Surgical approach**	
Open	28 (93.3%)
Laparoscopic/robotic	2 (6.7%)
**HIPEC chemotherapy**	
Cisplatin	13 (43.3%)
Cisplatin and mitomycin-C	1 (3.3%)
Mitomycin-C	12 (40.0%)
None	4 (13.3%)
**Peritoneal carcinomatosis index**	
Mean (std)	13.0 (10.5)
**Completeness of cytoreduction score**	
0	22 (75.9%)
1	4 (13.8%)
2	3 (10.3%)
**Neoadjuvant therapy**	
No	11 (36.7%)
Yes^c^	19 (63.3%)
**Neoadjuvant radiotherapy**	
No	27 (90.0%)
Yes	3 (10.0%)
**Adjuvant chemotherapy**	
No	16 (53.3%)
Yes^d^	14 (46.7%)
**Complications**	
No	24 (80.0%)
Yes^e^	6 (20.0%)
**RECIST response (N** = **19)**^f^	
Partial response	8 (42.1%)
Stable disease	6 (31.6%)
Progressive disease	5 (26.3%)

^a^Other histologies included colon (1), gastric (1), mesothelioma (2), leiomyosarcoma (1), and liposarcoma (1).

^b^Previous abdominal surgeries included appendectomy (9), hysterectomy (2), total abdominal hysterectomy with bilateral salpingo-oophorectomy (7), cholecystectomy (2), splenectomy (1), partial colectomy (1), Roux-en-Y gastric bypass (1), inguinal hernia repair (1), and prior CRS-HIPEC (3).

^c^Neoadjuvant chemotherapy regimens (N = 17) included carboplatin/paclitaxel (8), carboplatin/taxotere/bevacizumab (1), FOLFOX (1), FOLFIRI (1), CapeOx (2), FLOT (1), adriamycin/ifosfamide/mesna (1), and cisplatin/pemetrexed/ bevacizumab (2). In addition, one patient with gastric carcinomatosis received nivolumab, and one patient with sarcomatosis received selinexor. Thus, total patients who received some form of neoadjuvant systemic therapy was 19.

^d^Adjuvant chemotherapy regimens included carboplatin/paclitaxel (8), cisplatin/gemcitabine (1) FOLFOX (1), capecitabine/bevacizumab (1), cisplatin/pemetrexed (2), and ramucirumab/paclitaxel (1).

^e^Complications included wound infection (4), incisional hernia (1), and fascial dehiscence (1). None of these complications required re-operation.

^f^Response Evaluation Criteria in Solid Tumors (RECIST) was calculated for 19 patients who received neoadjuvant chemotherapy or other form of neoadjuvant systemic therapy.

For the remaining 26 patients (86.7%) who received HIPEC, cisplatin and/or mitomycin-C was perfused for 90 min. Three patients received neoadjuvant radiation for gastric adenocarcinoma (1), retroperitoneal dedifferentiated liposarcoma (1), and pelvic leiomyosarcoma originating from the left iliac vein (1). Of the 19 patients who received neoadjuvant therapy, 42.1% achieved a partial response (based on RECIST criteria), 31.6% had stable disease, and 26.3% had progressive disease. Additional information is provided in the footnotes of Table 1. The median follow-up period was 20.2 months. The median DSS was 25.2 months (95% CI 14.4, not reached). The 1-year and 2-year DSS rates were 0.91 (95% CI 0.68–0.98) and 0.56 (0.20–0.78), respectively.

A total of 6 patients (20%) had complications from CRS-HIPEC, with the most common being wound infection (4), followed by post-operative hernia (1), and fascial dehiscence (1). However, none of these complications were the result of performing the HIVM. The post-operative infection rate of 13.3% (4/30) is similar if not lower than the results of clinical trials of CRS-HIPEC^6,18–20^. No patients demonstrated adverse or allergic reactions to IV administration of fluorescein.

### HIVM vessel characteristics

At the time of the HIVM observation, patient heart rate and mean arterial blood pressure were recorded, which on average were 85.9 ± 13.8 beats per minute and 79.0 ± 9.4 mmHg, respectively. Tumor vessel and control vessel observations were feasible in all 30 patients. Thus, our primary objective was successfully completed for each patient in our cohort.

Table 2 shows the vessel characteristics obtained from the HIVM observations, analyzed by the overall cohort (n = 30), those who received neoadjuvant therapy (n = 19), and those who did not receive neoadjuvant therapy (n = 11). Similar numbers of tumor and control fields were observed among the cohort as a whole (*p* = 0.25). Statistically significant differences between the tumor and control fields were observed among all vessel measurements with the exception of mean non-functional vessel diameters. Interestingly, tumor-associated areas were observed to have a lower density of functional vessels (*p* = 0.0018), higher density of non-functional vessels (*p* < 0.0001), and higher proportion of non-functional vessels compared to non-tumor control areas (*p* < 0.0001). The mean diameter of functional vessels within tumor areas was also significantly smaller than the mean diameter of functional vessels within non-tumor areas (*p* < 0.0001). Conversely, the mean diameter of non-functional vessels was similar between the tumor and non-tumor areas (*p* = 0.15). Lastly, the mean blood flow velocity of functional vessels within tumor areas was significantly slower than the mean velocity of functional vessels within non-tumor areas (*p* < 0.0001).

**Table 2:** Comparison of tumor and non-tumor (control) vessel characteristics during HIVM observations.

Variable	Entire cohort (N = 30)		Neoadjuvant therapy (N = 19)		No neoadjuvant therapy (N = 11)	
	Mean (Std)	*p* value	Mean (Std)	*p* value	Mean (Std)	*p* value
**Number of observed fields**						
Control	8.8 (4.0)	0.25	9.4 (4.3)	0.22	8.2 (3.6)	0.63
Tumor	7.4 (5.2)		7.4 (4.7)		7.2 (6.1)	
**Density of functional vessels**						
Control	2.6 (0.77)	0.0018	2.8 (0.8)	0.015	2.4 (0.7)	0.07
Tumor	1.8 (0.9)		1.9 (0.9)		1.6 (1.0)	
**Density of non-functional vessels**						
Control	0.32 (0.40)	< 0.0001	0.3 (0.4)	< 0.001	0.4 (0.5)	0.002
Tumor	1.5 (0.75)		1.5 (0.7)		1.6 (0.8)	
**% Non-functional vessels**						
Control	11.2 (12.8)	< 0.0001	9.8 (11.5)	< 0.001	12.9 (14.5)	0.002
Tumor	46.0 (22.2)		42.4 (20.3)		50.8 (24.6)	
**Diameter of functional vessels (μm)**						
Control	32.3 (8.9)	< 0.0001	30.6 (6.5)	< 0.001	34.5 (11.2)	< 0.001
Tumor	16.4 (8.2)		17.2 (7.6)		15.3 (9.0)	
**Diameter of non-functional vessels (μm)**						
Control	12.1 (10.9)	0.15	11.5 (10.9)	0.35	12.9 (11.3)	0.30
Tumor	15.8 (7.4)		14.6 (6.9)		17.5 (7.9)	
**Velocity of functional vessels (μm/s)**						
Control	294.7 (163.3)	< 0.0001	328.7 (173.7)	< 0.001	246.7 (140.4)	0.06
Tumor	120.9 (71.7)		110.5 (70.1)		140.4 (75.3)	

Sub-analyses based on receipt of neoadjuvant therapy showed similar results, although for the no neoadjuvant therapy group, differences in the density of functional vessels and velocity of functional vessels did not reach statistical significance (*p* = 0.07 and 0.06, respectively). This was likely attributed to the smaller subset sample size for the no neoadjuvant therapy group (n = 11). However, the overall trends were similar.

**Figure 1:**
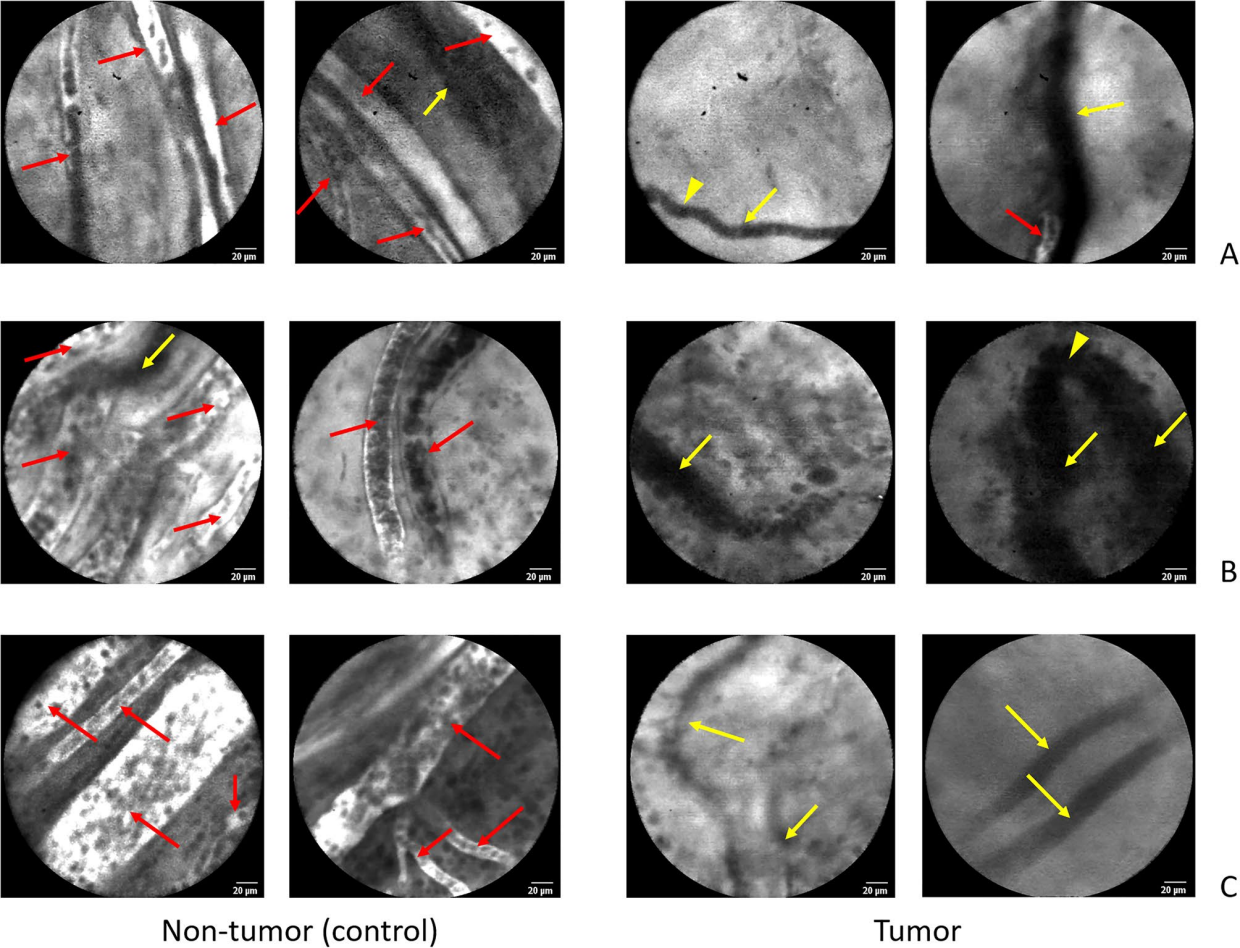
HIVM images of tumor and non-tumor vessels among individual. The right side panel shows examples of PC-associated tumor vessels from different histologies, including appendiceal adenocarcinoma (**A**), ovarian serous carcinoma (**B**), and mesothelioma (**C**). Yellow arrowheads highlight aberrantly arranged PC-associated vessel architecture (such as the acute hair-pin turn in part B), and yellow arrows highlight non-functional vessels as noted by the absence of fluorescein uptake. Non-tumor associated vessels from the same patients in the left side panel show normal, streamlined blood vessels, fewer non-functional vessels, and a higher number of functional (fluorescent) blood vessels. Red arrows highlight functional, normal blood vessels. Non-tumor areas were found to have some non-functional blood vessels, and conversely tumor areas were also found to have some functional blood vessels (red arrows).

**Figure 2:**
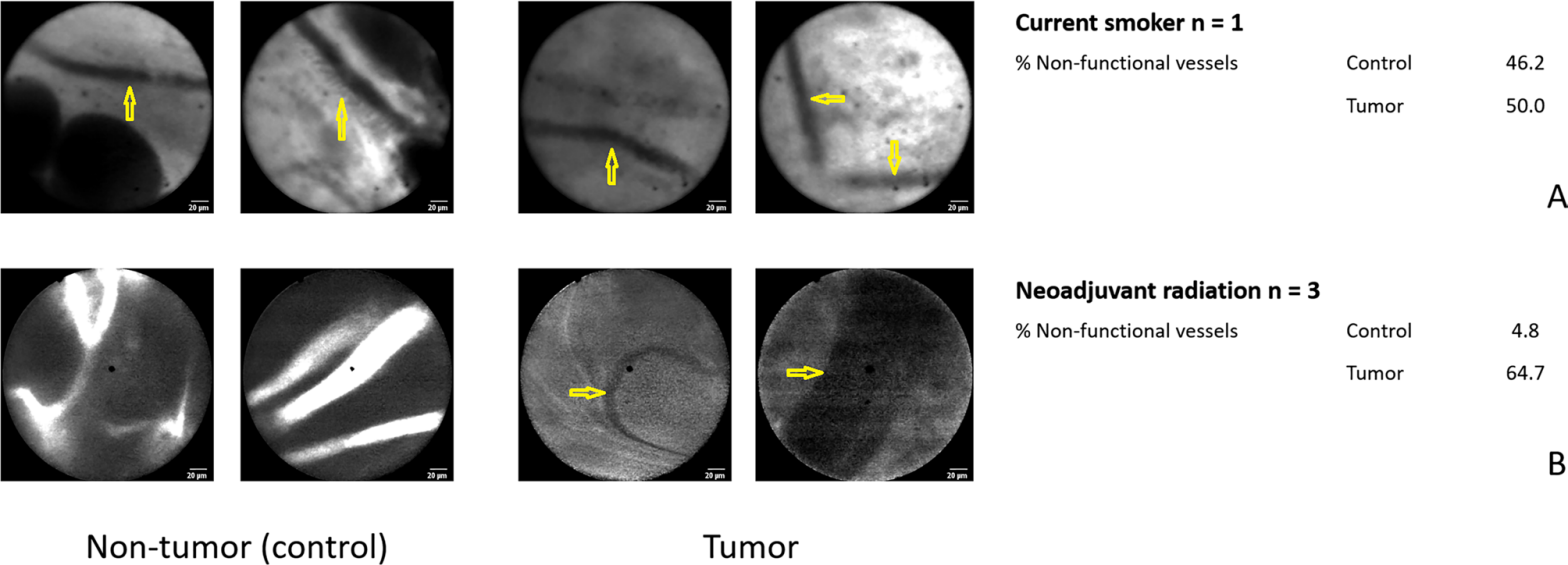
HIVM observations from non-tumor (left panel) and tumor (right panel) areas obtained from a current smoker (**A**) and from a patient who received pre-operative radiotherapy (**B**). There was a similar proportion of non-functional vessels between the tumor and non-tumor areas in the current smoker, suggesting pre-existing background changes in the microvasculature due to smoking use. In patients who received pre-operative radiotherapy (n = 3), there was a high proportion of non-functional vessels observed (64.7%). These findings suggest that radiation-induced changes had affected the tumor microvasculature.

Examples of real-time HIVM images of tumor and non-tumor vessels among individual patients are shown in Fig. 1. The right side panel shows examples of PC-associated tumor vessels from different histologies, including appendiceal adenocarcinoma (A), ovarian serous carcinoma (B), and mesothelioma (C). The aberrantly arranged PC-associated vessel architecture and presence of non-functional vessels (as noted by the absence of fluorescein uptake) are highlighted by yellow arrowheads and arrows, respectively. The rightmost panel of Fig. 1B demonstrates a classic example of a hair-pin turn in a non-functional vessel, which has been previously demonstrated by our group both in animal tumor models and in human subjects with melanoma^10,21^. In contrast in the left side panel, non-tumor associated vessels from the same patients show a high proportion of normal, streamlined blood vessels (red arrows) that demonstrate fluorescein uptake and a low proportion of non-functional vessels (yellow arrows). Figure 2 shows example HIVM observations from both non-tumor control (left panel) and tumor (right panel) areas obtained from a patient who was currently smoking (A) and from a patient who received pre-operative radiotherapy for a pelvic leiomyosarcoma (B). Interestingly, there was a similar proportion of non-functional vessels between the tumor and non-tumor areas in the current smoker (50.0% versus 46.2%, respectively). Patients who received pre-operative radiotherapy (n = 3), like the patient HIVM examples shown in Fig. 2B, had a high proportion of non-functional tumor vessels observed (64.7%).

### Vessel characteristics and outcomes among patients who received neoadjuvant therapy

Associations between HIVM PC-associated vessel observations and oncologic outcomes among patients who received neoadjuvant therapy (n = 19) were performed. Table 3 shows the univariate analysis between HIVM tumor vessel observations and RECIST response to neoadjuvant therapy, and Table 4 shows the univariate analysis between HIVM tumor vessel observations and DSS. No statistically significant associations were found between the HIVM vessel characteristics and RECIST response or DSS.

**Table 3:** Univariate analysis of tumor vessel characteristics with RECIST response among patients who had received neoadjuvant therapy (n = 19).

Variable	Odds ratio (95% CI)	*p* value
Density of functional vessels	0.95 (0.36, 2.51)	0.92
Density of non-functional vessels	0.46 (0.11, 1.85)	0.27
% Non-functional vessels	0.98 (0.94, 1.03)	0.40
Diameter of functional vessels	0.94 (0.84, 1.06)	0.32
Diameter of non-functional vessels	0.86 (0.71, 1.04)	0.11
Velocity of functional vessels	1.00 (0.99, 1.01)	0.98

**Table 4:** Univariate analysis of tumor vessel characteristics with disease-specific survival among patients who had received neoadjuvant therapy (n = 19).

Variable	Hazard Ratio (95% CI)	*p* value
Density of functional vessels	1.01 (0.30, 3.36)	0.98
Density of non-functional vessels	0.30 (0.07, 1.30)	0.11
% Non-functional vessels	0.97 (0.92, 1.02)	0.31
Diameter of functional vessels	0.97 (0.81, 1.16)	0.75
Diameter of non-functional vessels	0.98 (0.84, 1.15)	0.83
Velocity of functional vessels	1.00 (0.99, 1.01)	0.46

**Figure 3:**
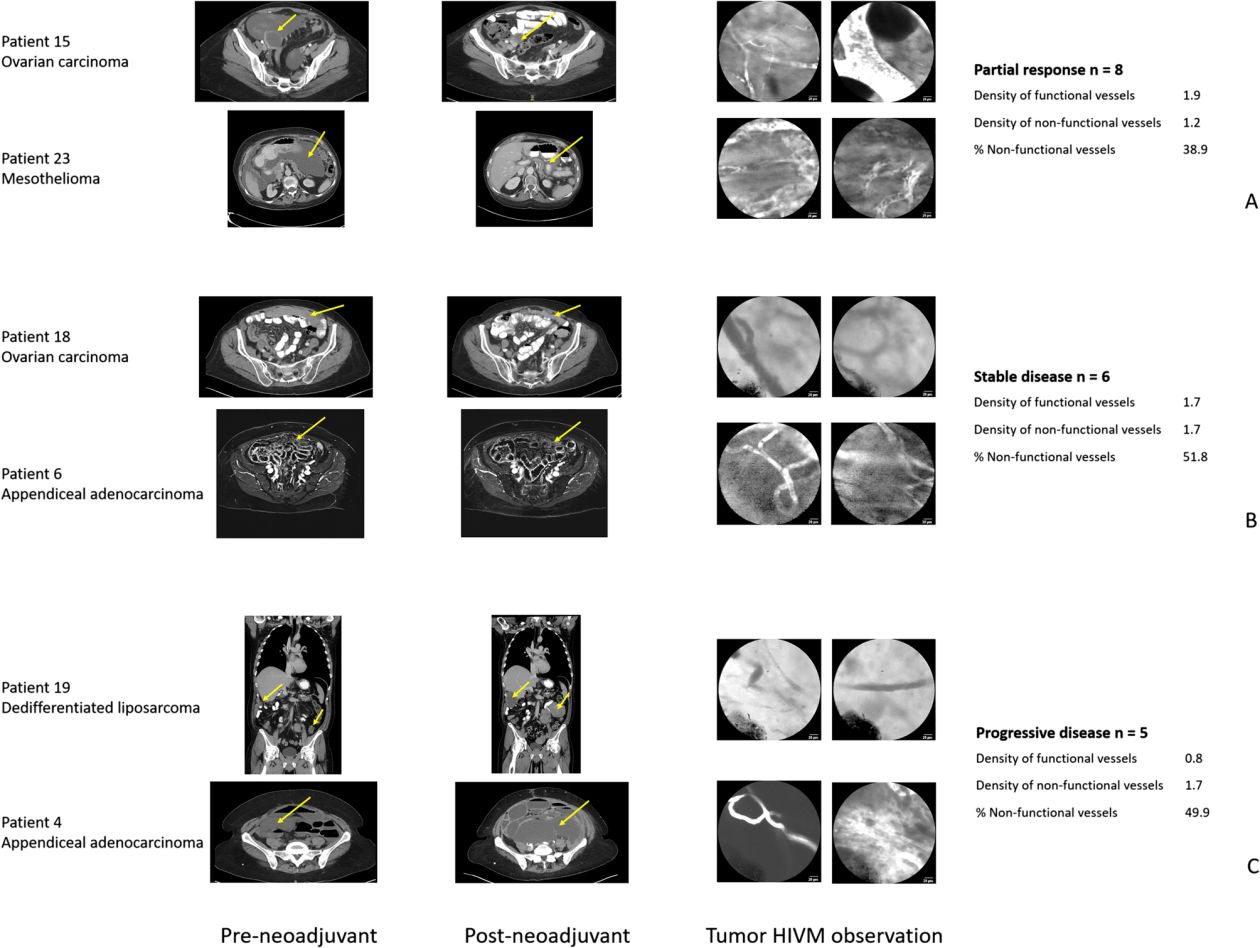
Examples of HIVM tumor observations for patients who had partial response (**A**), stable disease (**B**), and progressive disease (**C**) by standard RECIST criteria. The left side panels depict baseline imaging (CT scan or MRI) prior to neoadjuvant therapy. The middle panels depict follow-up imaging after neoadjuvant therapy just prior to undergoing CRS-HIPEC. Yellow arrows highlight the areas of disease. The right side panels depict HIVM PC-associated vessel observations. Patients who had achieved partial response had the highest density of functional vessels, lowest density of non-functional vessels, and the lowest proportion of non-function vessels when compared to patients who had stable disease or progressive disease. Although the subset analysis of patients who had neoadjuvant therapy did not show significant associations between RECIST response and tumor HIVM vessel characteristics, these individual patient observations provide early evidence that there may be a correlation between tumor response and tumor-associated vessels.

Although this pilot study did not show significant associations with tumor-associated vessels and outcomes, these were exploratory secondary outcomes and the sample size was not powered to detect such differences. However, among patients who received neoadjuvant systemic therapy (n = 19), we observed qualitative associations between RECIST response and tumor-associated vessel observations. Figure 3 shows examples of HIVM tumor observations for patients who had partial response (A), stable disease (B), and progressive disease (C). The left side panels depict baseline imaging (CT scan or MRI) prior to neoadjuvant therapy. The middle panels depict follow-up imaging after neoadjuvant therapy prior to CRS-HIPEC. The right side panels depict HIVM PC-associated vessel observations. Interestingly, patients who had achieved PR had the highest density of functional vessels, lowest density of non-functional vessels, and the lowest proportion of non-functional vessels when compared to patients who had SD or PD. A summary of the average vessel characteristics among patients with PR, SD, and PD are shown to the right of the HIVM images.

## Discussion

In this pilot study, we showed for the first time, that HIVM observations were feasible for patients with PC during the course of CRS-HIPEC. To date, there are a small number of reports demonstrating successful IVM techniques in the study of human cancers outside of hollow organs (GI tract, bladder)^10,22,23^. Similar to our previous study of HIVM observations of melanoma-associated vessels where up to 50% of vessels were non-functional^10^, PC-associated vessels also had a high rate of non-functionality (mean percentage of non-functional tumor vessels = 46.0%). In this study, we performed HIVM observations of non-tumor control areas as a comparison for tumor-bearing areas. Expectedly, we observed several differences between normal blood vessels and PC-associated vessels, reflecting what has been previously shown in animal models by our group and others^14,15,24^. Compared to the microvasculature in non-tumor control areas, PC-associated areas had on average a lower density of functional vessels, higher density of non-functional vessels, a higher proportion of non-functional vessels, smaller diameter of functional vessels, and slower blood flow velocity. These HIVM observations were consistent across the various tumor histologies that were included in the trial. However, some patient factors (smoking history) and treatment-related factors (neoadjuvant radiation) likely impacted these HIVM observations as well.

This study demonstrated several benefits of using HIVM in the study of PC. Unlike other live imaging techniques, such as CT or MRI, HIVM allowed for high magnification (100×) observations at the PC cellular level rather than at the organ level^14,25,26^. Individual microscopic vessels were observed using HIVM, leading to the data and images presented above and providing an in-depth view of the structure and function of tumor-associated vessels. Blood flow velocities were calculated using HIVM, and in this study PC-associated vessels were significantly lower than non-tumor control vessels. In addition, in contrast to the conventional pathologic analysis of fixed tissue sections ex vivo, HIVM allowed for the investigation of tumors and the tumor-associated microvasculature within the in vivo host environment. Our prior study of HIVM in melanoma showed that tumor-associated vessel diameters were larger than predicted from immunohistochemistry^10^, and thereby had lower wall shear stress, which could influence the delivery of systemically administered chemotherapeutic drugs or cell-based immunotherapies^27–29^. Interestingly in this study, the diameters of functional vessels within the tumor areas were on average smaller than the diameters of functional vessels within non-tumor areas. The smaller lumenal diameters of PC-associated vessels may have been due to structural differences in tumor neovascularization, which has also been shown to account for the aberrant organization of the tumor microvasculature^30,31^.

Our HIVM measurements of PC-associated vessels may have significant implications on the effectiveness of systemic drug delivery to target tumor. Many animal models have characterized several different structural and functional mechanisms by which tumor-associated vessels limit the delivery, and therefore efficacy, of systemically administered anticancer agents. The haphazard organization of tumor-associated vessels has been shown to correlate with poor response to anticancer treatments, such as anti-angiogenic agents, as well as worse survival outcomes^32–34^. As tumors grow, they often develop elevated interstitial pressure or hypertension that impedes afferent blood flow into the tumor^35–38^. This also results in increased efferent blood flow out of the tumor, which would further limit exposure time of systemic therapy within the tumor. The slower blood flow velocity observed with PC-associated vessels compared to normal vessels was consistent with the elevated interstitial hypertension of tumors. The increased proportion of non-functional vessels and decreased density of functional vessels within tumor areas were also consistent with decreased blood flow to tumor, which could be expected to result in decreased delivery of systemic therapy. The lack of fluorescein uptake within PC-associated non-functional vessels intuitively suggested that systemic therapy would also not be carried within those blood vessels. Taken together, these HIVM observations indicated that many PC-associated vessels would have diminished or no blood flow. Because the blood flow rate is directly proportional to vessel diameter and flow velocity (according to the equation: ¼ π d^2^ × v), our real-time HIVM measurements validated the presence of decreased blood flow in PC-associated vessels, consistent with several preclinical studies^16,39–42^.

As an exploratory component of our study, we sought to determine if these tumor-associated HIVM observations correlated to oncologic outcomes, including tumor response (RECIST) to neoadjuvant therapy and DSS. While some striking observations (Fig. 3) were made with regard to tumor response among individual patients with PR, SD, or PD, the overall analysis did not show any statistically significant associations between HIVM tumor vessel measurements and tumor response. The lack of statistical significance was likely due to this study being underpowered to detect a significant difference in RECIST response for a specific tumor histology. Although our study enrolled 30 patients, the histologies varied among appendiceal cancer (40%), ovarian cancer (40%), and a heterogeneous mixture of other primary malignancies (20%). In addition, only 19 (63.3%) patients received neoadjuvant systemic therapy and were included in this outcome analysis. We intend to address this limitation through our larger, ongoing HIVM clinical trial, Intravital Microscopy in Human Solid Tumors (ClinicalTrials.gov identifier: NCT03823144)^43^. The long-term goal of these clinical trials is to determine if HIVM can serve as an imaging biomarker for surface malignancies. HIVM may not only be applied to patients who are undergoing surgery, but may potentially be used for patients with surface malignancies who are receiving systemic therapy, such as cutaneous malignancies or inflammatory breast cancer^44,45^. These surface malignancies easily lend themselves to investigation with HIVM. In addition, some systemic agents (for example doxorubicin) possess an intrinsic autofluorescence, which may be used to quantitate penetration into the tumor target via HIVM^46,47^.

We acknowledge that there are additional limitations to this study. The HIVM observations were limited to two normal (control) areas and two gross tumor-bearing areas. These areas were randomly chosen at the time of CRS-HIPEC. However, many patients, particularly those with higher PCI scores, had more than two areas of carcinomatosis. While HIVM observations of additional tumor areas could have been performed, each observation required at least 2–3 min per area. This resulted in an HIVM observation time of approximately 8–12 min per subject. Thus, it was essential to establish a balance between HIVM data acquisition and prolongation of the surgical and anesthesia time for the patient. The CRS-HIPEC procedure already takes an average of 8 h at our institution when performed open and 6 h when performed using a minimally invasive approach^48,49^, so additional time allocated to HIVM observations further increased the length of the entire procedure. While we assumed that the selected tumor areas were representative of the PC as a whole, this was not proven by our study design. Furthermore, the microscope does not have the capability to distinguish between small blood vessels and lymphatic vessels. Thus, for visualized non-functional vessels (that were not observed to have dye uptake or red blood cells), some of these structures may have represented lymphatics as opposed to blood vessels. Because lymphatics would not be expected to delivery systemic therapy to tumor, the proportion of non-functional vessels that were in fact lymphatic vessels may have implications on systemic drug delivery and outcomes to neoadjuvant therapy. In addition, image stabilization sometimes proved difficult because the imaging probe had to be manually held in place and because respirations also caused fluctuations in vessel visualization. However, these limitations were offset by briefly holding respirations (for a maximum of 30 s) and through use of stabilization software (IC Viewer, Mauna Kea Technologies, Paris, France) that provided the ability to analyze the acquired videos frame by frame (averaging 9 frames per second). Thus, high quality images and measurements were still obtainable, even when they were performed through a minimally invasive approach.

Despite these limitations, HIVM observations were highly feasible (100%) in our cohort of patients with PC of varying histologies at the time of CRS-HIPEC. Important characteristics of tumor-associated microvasculature as compared to normal blood vessels were identified, including a higher density and proportion non-functional vessels, smaller tumor vessel diameters, and slower blood flow velocity in tumor areas. While this study did not find statistically significant associations between these HIVM observations and oncologic outcomes, it has further built on the growing body of evidence that HIVM can be used as a tool for patients with cancer, specifically PC in this trial. Ongoing and future planned clinical trials with larger cohorts of patients have a high likelihood of further determining the significance of HIVM tumor vessel observations in cancer treatment.

## Patients and methods

### Patient selection

This was a single center, nonrandomized pilot study of HIVM observation in subjects with PC undergoing CRS-HIPEC. Target accrual was 30 patients based on our previous pilot study in melanoma^10^, and the diverse histologies presenting as carcinomatosis. Patients with measurable tumor on the peritoneal surface by direct visualization eligible for CRS-HIPEC were evaluated for entry into clinical trial PR17-009823 Intravital Microscopy (IVM) in Patients with Peritoneal Carcinomatosis (PC) at Mayo Clinic Florida, Jacksonville, Florida (ClinicalTrials.gov identifier: NCT03517852, date of first registration 08/05/2018)^50^. This trial was approved by the Institute Review Board of Mayo Clinic, and the protocol can be accessed at https://clinicaltrials.gov/ct2ratehow/NCT03517852. Additional inclusion and exclusion criteria are listed in Supplementary Table 1. The trial was performed in accordance with all relevant guidelines and regulations, and informed consent was obtained from all participants. Specifically, this trial conformed to the CONSORT 2010 guidelines for reporting a pilot or feasibility trial. This trial was also registered in the ISRCTN registry, a WHO International Clinical Trial Registry Platform (registration date: 25/11/2020, registration number: 10645335, “Using microscopes to observe tumor vessels”).

Eligible patients who provided informed consent were enrolled in the protocol. Enrollment was performed by Drs. Gabriel, Bagaria, Robertson or Dinh. Recorded information included demographic data (age, sex, BMI, race, history of smoking, history of diabetes, prior abdominal surgery), tumor-specific data (tumor histology and subtype, grade, peritoneal carcinomatosis index or PCI score, completeness of cytoreduction or CC score, primary versus recurrent diagnosis), and treatment-related variables (receipt and type of neoadjuvant, intraoperative, and adjuvant chemotherapy or other systemic therapy; receipt of neoadjuvant radiotherapy, radiographic response to neoadjuvant therapy as measured by standard RECIST criteria, surgical approach, and complications from CRS-HIPEC). Response Evaluation Criteria in Solid Tumors (RECIST) has been used to evaluate response to systemic therapy in peritoneal malignancies^51–53^.

The fluorescein skin prick test was performed as previously described in NCT01886235 A Pilot Study of Feasibility of Performing Intravital Microscopy in Melanoma Patients^10^. The reported risk of anaphylactic reaction to fluorescein was very low (1 in 222,000)^54,55^, but as this study was performed for investigational purposes, subjects were deemed ineligible if they had a positive fluorescein skin prick test.

### Surgical technique

The technique of CRS-HIPEC performed at our institution has been previously described^56^. In brief, inspection of the abdomen was performed through a either a diagnostic laparoscopy or mini-laparotomy to determine the feasibility of obtaining a CC score of 0^57^. Each patient was assigned a PCI score^57^. Patients with low PCI (≤ 5) were candidates for minimally invasive (MIS) surgery (laparoscopic or robotic-assisted). Standard CRS for all patients of any histology included both greater and lesser omentectomies, resection of the falciform ligament, and bilateral salpingo-oophorectomy (BSO) in female patients. Additional sites of gross disease were cytoreduced, including visceral resections, resection of previous scars, and peritonectomies, or fulgurated with Bovie electrocautery or argon beam coagulation as indicated at the time of surgery. Following CRS, closed HIPEC was initiated. For tumors of appendiceal or colorectal origin, mitomycin C (30 mg fixed dose) was used. For tumors of ovarian origin, cisplatin (100 mg/m^2^) was used. Perfusion was initiated when the outflow catheter temperature reached 41 °C. Temperature was maintained between 41 and 43 °C, and the perfusion time was 90 min.

### Microscope

We used the ultra-high definition (UHD) probe-based confocal laser endomicroscopy device (pCLE; Gastroflex, Cellvizio System, Mauna Kea Technologies, Paris, France) to obtain the HIVM observations. This microscope allows for imaging to a depth of 10 µm (μm) with 1–2 μm resolution at an average acquisition rate of 9 frames per second. Patient tumor-associated vessels were observed at 100 × magnification, which allowed for observations at the capillary level. This technology has previously been used by our group and others to perform real-time observations of GI malignancies and pre-malignancies through an endoscopic approach^58–60^. Although this device was initially developed for endoscopic observations of the GI tract (esophagus, stomach, biliary ducts, colon, and rectum), this device also allows HIVM observations of PC through an open or MIS approach. Videos were obtained in a proprietary format (.mkt) and also converted to a universal (.avi, .mpeg, or .wmv) format for analysis. Offline quantification of vessel characteristics was performed using the Mauna Kea Technologies IC-Viewer. All images/videos were stored on a password-protected institutional hard drive.

### Intravital microscopic observations in patients

Prior to CRS-HIPEC, HIVM observations were performed on two separate tumor-bearing areas and two separate non-tumor bearing control areas. These areas were separated by a distance of at least 10 cm (cm). We selected the tumor areas based on the highest amount of gross tumor burden that was visualized. The pCLE was appropriately sterilized and covered with a sterile sleeve as an additional precaution. A small slit was cut in the sleeve to allow the tip of the pCLE to directly observe the surface vessels. Prior to performing the HIVM observation, the patient heart rate and mean arterial blood pressure (MAP) were recorded.

One milliliter (ml) of 5% fluorescein (Akorn Inc., Lake Forest, Illinois) was injected via a peripheral IV catheter. Imaging acquisition occurred 15–20 s after fluorescein injection. Once the microscope was directly overlying the exposed tumor tissue, the fluorescent light source and image acquisition software were activated. The microscope was held by the operating surgeon over the selected areas of observation. In cases of MIS CRS-HIPEC, the pCLE was inserted through the laparoscopic/robotic trocars and stabilized with MIS graspers. To facilitate stabilization of the HIVM observations, respirations were temporarily held by anesthesia for a maximum of 30 s per observed area.

Tumor vessels were clearly identified by their aberrant architecture, absence of dye uptake, and non-uniform blood flow patterns. HIVM observations focused on two separate tumor areas for approximately 2–3 min per area. These were surface areas that contained gross tumor deposits and included the peritoneal lining or visceral peritoneum (small bowel, colon, mesentery). Within each area, multiple fields (up to 20 distinct fields) were observed over an area of approximately 2 square cm during each observation period. Similarly, two areas which did not have any gross tumor were observed as controls for non-tumor associated vessels. Control observations were also performed over several fields per area and consisted of approximately 2–3 min per area. In total, the HIVM observations took approximately 8–12 min per patient (including 2 control and 2 tumor areas). A second dose of fluorescein (1 ml 5%) was given as needed to complete the HIVM observations. This second dose provided additional contrast to facilitate visualization of the vessels after the first dose of fluorescein diffused out into the surrounding tissues. An observation was completed when fluorescein was noted to have extravasated out of tumor/control vessels into the surrounding background tissue. Tumor and control area observations were alternated to minimize differences in background fluorescein extravasation and attempt to maintain similar levels of contrast.

### Post hoc analyses

The primary objective was to determine the feasibility of performing HIVM in patients with PC during standard course of CRS-HIPEC. The secondary objectives were to determine the associations between (1) HIVM tumor vessel observations and response to neoadjuvant therapy and (2) HIVM tumor vessel observations and oncologic survival outcomes. Feasibility was assessed by the ability to quantitate the following primary outcome measures: (1) vessel identification per high power field, (2) vessel density (number of vessels divided by number of fields), (3) fluorescein uptake, and (4) blood flow velocity.

Analysis of the HIVM observations was performed as previously described by our group (refer to Fig. 2 of reference 10)^10,21^. Abnormal blood vessels were characterized by predefined characteristics as listed in Supplemental Table 2. Prior to the analysis, HIVM observations were de-identified to minimize bias. The number of observation fields (for both tumor and control areas) was recorded. Tumor-associated and control vessels were characterized morphologically. A single vessel was described as beginning at a branch point and continuing to the next branch point. To be considered measurable, vessels had to be at least 100 μm in length with no branch points. Vessels were considered functional if fluorescein uptake was identified or if blood flow within the vessel could be visualized. In contrast, vessels were considered non-functional if there was no fluorescein uptake or no blood flow was visualized. Total numbers of functional and non-functional vessels were recorded. Vessel density (both functional and non-functional) was established by dividing the number of vessels by the number of fields of observation per area (control or tumor). The percentage of non-functional vessels per area (tumor or control) was calculated by dividing the number of non-functional vessels by the total number of vessels observed (# non-functional vessels / # non-functional vessels + # functional vessels) times 100.

IC-Viewer software (Mauna Kea Technologies, Paris, France) was used to measure vessel diameter (d) at the vessel’s largest width. Blood flow velocity (v) was evaluated by determining the time that distinct features in an observed vessel would take to travel a known distance. Velocity was calculated by dividing the measured distance by the time taken to travel that distance, and then averaging these values for at least 3 points per vessel. No velocities were calculated for non-functional vessels as by definition, these vessels did not support any blood flow.

### Statistical analyses

Demographic and clinical characteristics were summarized using the mean and standard deviation (std) for continuous variables, and using frequencies for categorical variables. Response (RECIST) was summarized using frequencies. Disease-specific survival (DSS) was summarized using standard Kaplan–Meier methods. The 1- and 2-year survival rate and median survival were also reported. Vessel characteristics (diameter, density, and velocity) were summarized using mean and std. The two-sided, paired t test was used to make comparisons between the control and treatment groups. A univariate logistic regression model was constructed with response as the outcome and demographic, clinical, and vessel variables as the separate predictors. This was performed for patients who had received neoadjuvant therapy (n = 19). Odds ratios (OR) with 95% confidence intervals (CI) and the corresponding *p*-value were reported for each variable. A univariate Cox regression model was constructed with DSS as the outcome and demographic, clinical, vessel, and RECIST response variables as the separate predictors. Hazard ratios (HR) with 95% CI and the corresponding *p*-value were reported for each variable. All analyses were conducted in SAS v9.4 (Cary, NC) at a significance level of 0.05.

## Supplementary Information

Supplementary Information.

## Abbreviations

BMI: Body mass index
CC: Completeness of cytoreduction
CI: Confidence interval
CRS: Cytoreductive surgery
CT: Computerized tomography
d: Diameter
DPAM: Disseminated peritoneal adenomucinous
DSS: Disease-specific survival
GI: Gastrointestinal
GU: Genitourinary
HIPEC: Hyperthermic intraperitoneal chemotherapy
HIVM: Human intravital microscopy
HR: Heart rate
IV: Intravenous
IVM: Intravital microscopy
LAMN: Low grade appendiceal mucinous neoplasm
MAP: Mean arterial pressure
MIS: Minimally invasive surgery
MRI: Magnetic resonance imaging
OR: Odds ratio
PC: Peritoneal carcinomatosis
PCI: Peritoneal carcinomatosis index
pCLE: Probe-based confocal laser endomicroscopy device
RECIST: Response Evaluation Criteria in Solid Tumors
std: Standard deviation
UHD: Ultra-high definition
v: Velocity
VEGF: Vascular endothelial growth factor
