# Supplementary material for: A pilot trial of intravital microscopy in the study of the tumor vasculature of patients with peritoneal carcinomatosis
Source: Sci Rep. 2021 Mar 2;11:4946. doi: 10.1038/s41598-021-84430-3 (PMC7925603; doi:10.1038/s41598-021-84430-3)
Supplement: Supplementary file 1 — Supplementary Information. [file 41598_2021_84430_MOESM1_ESM.pdf]

Intravital microscopy in the study of the tumor vasculature of patients with peritoneal carcinomatosis

Running head: Tumor vessels in peritoneal carcinomatosis

Emmanuel M. Gabriel MD, PhD, FACS[1], Minhyung Kim MD[2], Daniel T. Fisher PhD[2], Catherine Mangum MD[1], Kristopher Attwood PhD[3], Wenyan Ji MS[3], Debabrata Mukhopadhyay PhD[4], Sanjay P. Bagaria MD, FACS[1], Matthew W. Robertson MD[5], Tri A. Dinh MD[5], Keith L. Knutson PhD[6], Joseph J. Skitzki MD, FACS[7], and Michael B. Wallace MD, MPH[8]

1. Department of Surgery, Section of Surgical Oncology, Mayo Clinic, Jacksonville, FL
2. Department of Immunology, Roswell Park Comprehensive Cancer Center, Buffalo, NY
3. Department of Biostatistics, Roswell Park Comprehensive Cancer Center, Buffalo, NY
4. Department of Molecular Biology, Mayo Clinic, Jacksonville, FL
5. Department of Gynecological Oncology, Mayo Clinic, Jacksonville, FL
6. Department of Immunology, Mayo Clinic, Jacksonville, FL
7. Department of Surgical Oncology, Roswell Park Comprehensive Cancer Center, Buffalo, NY
8. Department of Gastroenterology, Mayo Clinic, Jacksonville, FL

Corresponding Author:
Emmanuel M. Gabriel, MD, PhD, FACS
Assistant Professor of Surgery
Department of Surgery
Section of Surgical Oncology
Mayo Clinic Florida
4500 San Pablo Road
Jacksonville, FL 32224
Telephone: 904-953-2523
Email: Gabriel.Emmanuel@mayo.edu

Supplementary Tables

Supplementary Table 1. Inclusion and Exclusion Criteria

**Inclusion Criteria**
1. Age ≥ 18 years of age.
2. ECOG Performance Status of ≤ 2.
3. Measurable tumor on the peritoneal surface by direct visualization requiring surgical resection.
4. PC that meets indications for CRS-HIPEC. Sites of origin for tumor include appendix, colon, stomach, and primary peritoneal surface malignancies such as peritoneal mesothelioma. Sarcomatosis was also accepted.
5. Subject or legal representative must understand the investigational nature of this study and sign an Institutional Review Board approved written informed consent.
6. Subject must have a skin prick test pre-operatively (at the time of the preoperative visit and after signing the informed consent) to determine any sensitivity to fluorescein.
**Exclusion Criteria**
1. Uncontrolled illness including (but not limited to) active infection, symptomatic CHF, unstable angina, severe psychiatric illness, or extreme social situations that do not permit participation.
2. Renal dysfunction as defined as a GFR < 45.
3. Liver dysfunction as defined by Child-Pugh score > 5 or LFT's 1.5x above normal range.
4. Known allergy to fluorescein or a positive skin prick test to fluorescein.
5. Pregnant or nursing female subjects, determined preoperatively with a urine pregnancy test.
6. Unwilling or unable to follow protocol requirements.
7. Any condition that excludes CRS-HIPEC as the standard of care (e.g. high disease burden where alternative treatments like systemic chemotherapy would be preferred).

Supplementary Table 2. Definitions of abnormal blood vessels.

**Abnormal vessel characteristics**
1. Aberrant vessel structure compared to normal tissue, which includes tortuosity of the vessel wall or a closed loop formation.
2. Aberrant vessel branching patterns compared to normal tissue, which includes erratic and disorganized branching points.
3. Blood vessel density that is less than or greater than one standard deviation of the blood vessel density observed in normal tissue (non-tumor bearing area).
4. Inability to detect flow or intravital dye within a vessel.
5. Average blood flow rate through observed vessel that is less than or greater than one standard deviation of the average blood flow rate through a normal vessel.
